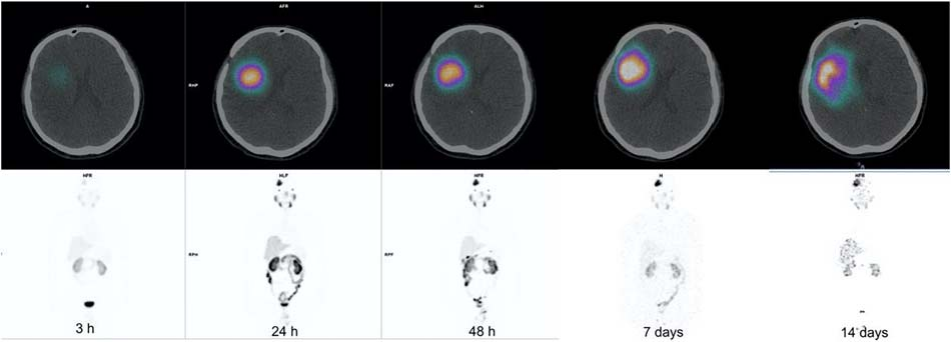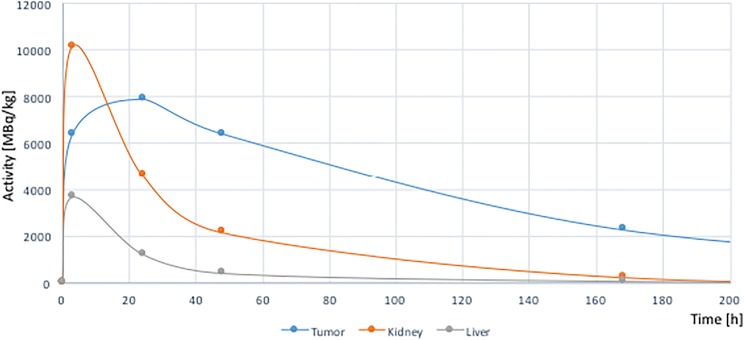# Tumor uptake in glioblastoma multiforme after IV injection of [^177^Lu]Lu-PSMA-617

**DOI:** 10.1007/s00259-020-04715-z

**Published:** 2020-02-10

**Authors:** Jolanta Kunikowska, Ingeborga Charzyńska, Radosław Kuliński, Dariusz Pawlak, Michał Maurin, Leszek Królicki

**Affiliations:** 1 Department of Nuclear Medicine, Medical University of Warsaw, ul. Banacha 1 a, 02-097 Warsaw, Poland; 2 Radioisotope Centre POLATOM, National Centre for Nuclear Research, Otwock-Świerk, Poland

Glioblastoma multiforme (GBM) is the most malignant primary brain tumor with limited treatment options in case of recurrence. Prostate-specific membrane antigen (PSMA) expression was demonstrated in microvascular endothelium of GBM and in vivo confirmed by [^68^Ga]Ga-PSMA-11 PET/CT in primary as well in recurrence tumor [1–4]. This knowledge opens a new way for targeted, PSMA-based treatment. However, it was suggested that uptake not in tumor cells, but in microvascular endothelium, could be characterized by quick washout.

A 54-year-old man 3 years after primary treatment including surgery and chemo-radiotherapy, with a recurrence of GBM revealed in the follow-up MRI, was referred for [^68^Ga]Ga-PSMA-11 PET/CT. The image demonstrated increased, homogenous uptake in the right parietal mass (SUVmax 10.3) with the tumor-to-liver ratio of 1.8. The patient was disqualified from surgery and radiotherapy and refused chemotherapy. [^177^Lu]Lu-PSMA-617 treatment was performed (8.39 GBq) with a dosimetry study [5].

The post-therapy images showed increased uptake in tumor mass during the first 24 h, with slow, decreased accumulation up to 14 days. The uptake in normal organs increased up to 3 h post injection and rapidly decreased in the next days (Figs. 1 and 2). The absorbed dose for the tumor was 14.07 Gy, kidney 0.14 Gy, liver 1.67 Gy, and for whole body 0.49 Gy. To our knowledge, dosimetry study in the treatment of GBM with [^177^Lu]Lu-PSMA-617 has not been previously reported. The post-therapy images proved the possibility of targeted therapy with α/β-emitters with no quick as postulated washout in the tumor.